# Patterns of Traditional Chinese Medicine Diagnosis in Thermal Laser Acupuncture Treatment of Knee Osteoarthritis

**DOI:** 10.1155/2013/870305

**Published:** 2013-08-28

**Authors:** Lizhen Wang, Fan Wu, Ling Zhao, Haimeng Zhang, Xueyong Shen, Yi Huang, Lixing Lao

**Affiliations:** ^1^Shanghai University of Traditional Chinese Medicine, 1200 Cailun Road, Shanghai 201203, China; ^2^Center for Integrative Medicine, University of Maryland, School of Medicine, 520 West Lombard Street, East Hall, Baltimore, MD 21201, USA; ^3^Department of Mathematics and Statistics, University of Maryland, Baltimore County (UMBC), 1000 Hilltop Circle, Baltimore, MD 21250, USA; ^4^School of Chinese Medicine, The University of Hong Kong, 10 Sassoon Road, Pokfulam, Hong Kong, China

## Abstract

Knee osteoarthritis (OA) manifests with pain, joint stiffness, and limited function. In traditional Chinese medicine, knee OA is differentiated into three patterns: *yang* deficiency and cold coagulation, kidney deficiency, and blood stasis. The objective of this study was to determine whether *yang* deficiency cold coagulation patients respond better to thermal laser acupuncture treatment than do non-*yang* deficient patients. Fifty-two patients with OA were allocated to group A (*yang* deficient, *n* = 26) or B (non-*yang* deficient, *n* = 26). All patients received a 20-min thermal laser acupuncture treatment at acupoint Dubi (ST 35) three times a week for two weeks and twice a week for another four weeks. Outcome assessments were performed immediately after the first treatment, and at weeks 2, 6, and 10. Group A function scores were significantly better than those of Group B at weeks 2 (*P* = 0.049), 6 (*P* = 0.046), and 10 (*P* = 0.042), but no significant differences were found between the two groups in pain and stiffness scores at any time point. No significant adverse effect was observed. The combined 10.6 **μ**m–650 nm laser treatment might be most beneficial to *yang* deficiency cold coagulation knee OA patients, particularly in improving function.

## 1. Introduction

Osteoarthritis (OA) manifests clinically as chronic joint pain, stiffness, and swelling accompanied by dysfunction. The knee is the joint most commonly involved, and knee OA is a chronic disease [1]. With aging, the incidence of the disease tends to increase, and pain and dysfunction of the knee can seriously affect life activities [2, 3]. No conventional cure is currently available, which makes knee OA a leading cause of pain and disability worldwide [4]. Pathological changes include loss of local articular cartilage and new bone formation at joint margins where destructive bone loss occurs [4]. Conventional therapies, usually NSAIDs, mainly are used to manage symptoms such as joint pain, improve function, delay the progression of joint damage, and improve patient quality of life [5]. These often have undesirable side effects such as gastrointestinal disorders, making treatment unsustainable [6]. 

Studies have shown acupuncture is successful in treating joint pain, including knee OA [7]. In Traditional Chinese Medicine (TCM) theory, chronic knee OA is considered to be mainly due to kidney deficiency, blood stagnation, and the retention of damp cold in the knee. Moxibustion, part of traditional acupuncture treatment, involves the burning of an herb (*Artemisia argyi*) at the site of acupoints; it is commonly used in treating *arthritis* particularly joint pain caused by cold [8–12]. However, the use of this technique is limited, particularly in the West, because of the smoke and inconvenience of the procedure, and caution is needed to prevent burning the patient's skin.

In recent years, lasers with red and near-infrared wavelengths such as the 650 nm laser have been widely used in clinical settings [13–17]. Several investigators report successful treatment of chronic pain, including knee OA, with these devices [18–23]. We recently developed an appliance that combines laser wave lengths of 10.6 *μ*m and 650 nm to mimic the effects of moxibustion and acupuncture: these wave lengths produce both the needle-like effect of acupuncture and the thermal effect of moxibustion [22, 23]. Because of the nature of the device, we hypothesized that it might be most effective for reducing pain and improving function in the cold syndrome of knee OA. We conducted a patient-centered, nonrandomized outcome effectiveness study to compare the response of *yang* deficiency cold coagulation pattern knee OA patients to 10.6 *μ*m–650 nm combined laser irradiation with that of OA patients with other TCM patterns. 

According to TCM theory, knee OA symptoms are differentiated into (1) *yang deficiency cold stagnation*, (2) *kidney essence deficiency*, and (3) *blood stasis and collateral stagnation* [24]. The main symptoms of *yang deficiency cold stagnation* are pain, stiffness, and impaired function in the knee joint, worse with cold, and alleviated by warmth. The patient often feels cold, fatigue, and heaviness in the limbs. The tongue is pale with white coating; the pulse is deep, thready, and slow. The main symptoms of *kidney essence deficiency* are limited range of motion, dizziness, and tinnitus. The tongue is pinkish with a thin coating; the pulse is thready. The main symptoms of *blood stasis and collateral stagnation* are fixed pain, limited range of motion, dark face, and purplish lips. The tongue is purple and dark; pulse is deep and thready. Of the three TCM patterns, yang deficiency cold stagnation is a cold syndrome and can be alleviated by a warm therapy.

In Chinese acupuncture practice, “warming needles,” in which moxa wool is burned on the handle of an acupuncture needle, is often used on patients with cold syndrome TCM patterns [25, 26]. The thermal effect of the combined 10.6 *μ*m–650 nm laser mimics the warming needle in that the 10.6 *μ*m CO_2_ laser wavelength has a thermal effect while the 650 nm laser wavelength has a needle-like effect. The purpose of the present study was to determine the usefulness of TCM pattern diagnosis in the effectiveness assessment of this laser treatment and to see whether thermal laser irradiation would be more effective on *yang* deficiency cold coagulation knee OA.

## 2. Materials and Methods

### 2.1. Patient Recruitment

The study was conducted from July 2007 to February 2009 in out-patient settings at two research centers in Shanghai, China: Shuguang Hospital affiliated to Shanghai University of Traditional Chinese Medicine and First People's Hospital affiliated to Shanghai Jiao Tong University. The research protocol was approved by the Institutional Review Board of Shuguang Hospital affiliated to Shanghai University of Traditional Chinese Medicine (no. 2007-N019-01). Each subject provided informed consent.

We recruited subjects by advertising in local newspapers. Interested subjects contacted the study coordinator, who conducted initial telephone screening. Potential subjects then received on-site clinical assessments in the hospital after getting a full description of the study and giving informed consent. 

Patients who met the inclusion criteria were included for the trial: male or female, between 50 and 75 years old, diagnosed with knee OA as defined by the American College of Rheumatology (ACR) [24] such as radiographic evidence of at least one osteophyte at the tibiofemoral joint, (Kellgren-Lawrence grade ≥2), moderate or greater knee pain on most days (>50%) during the previous month, and willingness to sign the consent form. Exclusion criteria were presence of serious medical conditions such as kidney or liver disease or deep vein thrombosis that precluded participation in the trial, intra-articular corticosteroid or hyaluronate injections, knee surgery during the past 6 months, participation in other clinical trials within the past six months, and travel or other plans that would interfere with participation in the entire six-week study. Patients were allowed to maintain their baseline pain medications during the course of experiment, and any changes in medication were documented.

### 2.2. Study Protocol

This was a nonrandomized outcome effectiveness study comparing the response to 10.6 *μ*m–650 nm combined laser irradiation treatment of *yang* deficient patients (Group A) to that of non-*yang* deficient patients (Group B). After subjects met the inclusion/exclusion criteria, one experienced TCM doctor carried out a pattern differentiation on each; based on the differentiations, we recruited 26 patients to each group. The *yang* deficiency cold coagulation pattern was defined as knee pain, aversion to cold in the joint, alleviation of symptoms by warmth, and aggravation by cold [27]. 

#### 2.2.1. Laser Device

**Figure 1:**
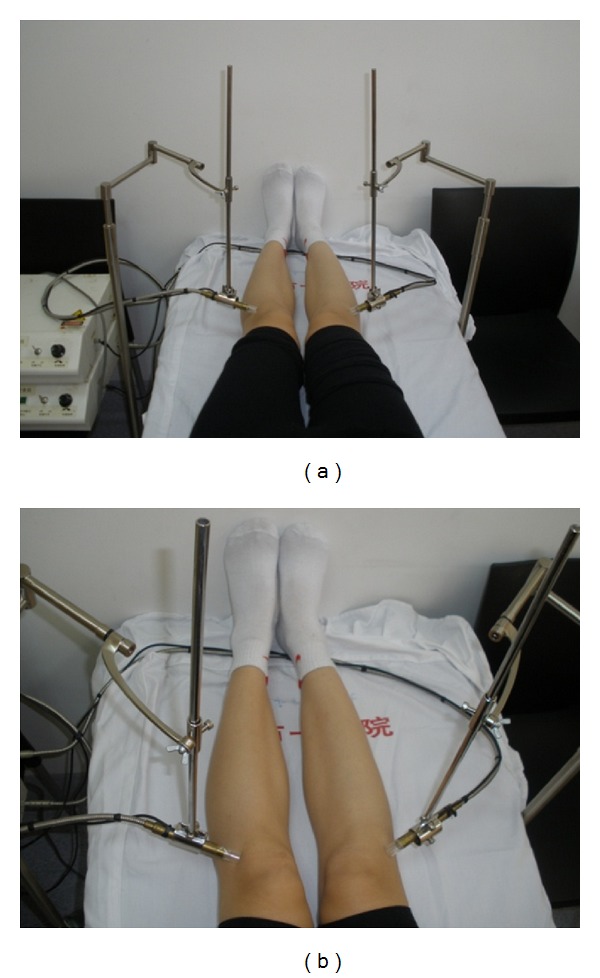
(a) Laser treatment was performed bilaterally. (b) Laser tips were placed at acupuncture point ST35 (Dubi).

The features of the device and the method of operation have been previously reported [22, 23]. In brief, 10.6 *μ*m–650 nm combined laser irradiation was generated by a semiconductor laser with a 0.65–0.66 *μ*m-long wave transmitted by quartz-glass light fibers (36 mW) and a CO_2_ laser with 10.6 *μ*m-long wave by a silver halide light fiber (200 mW). Output was set in accordance with the safety criteria of Shanghai Enterprises (Q/KYD012-2004). The output was transmitted to a combined laser tip that irradiated the skin with a single beam 2 mm in diameter. A 2 cm distance between the laser tip and the skin was maintained by a plastic tube, 1 cm in diameter and 2 cm in length, mounted on the tip of the device (Figure 1).

#### 2.2.2. Intervention

Laser treatments were performed by trained technicians blinded to the group allocation. During treatment, the room was maintained at a constant 20°C. The patient was instructed to lie supine for 5 min before treatment. The laser tip was placed perpendicularly on the affected knee, or on both knees if both were affected, at acupuncture point ST35 (Dubi) which is located in the depression on the lateral side of the patella and the patellar ligament [28] (Figure 1). ST 35, called Dubi or Xiyan (“eye of the knee”), is commonly used in clinical trials as a major local point for treating knee-related disorders [29–31]. The device was activated for 20 min, and the patient received 10.6 *μ*m laser energy of 120 J and 650 nm laser energy of 43.2 J at each session. Patients were treated three times a week for the first two weeks and twice a week for the next four, a total of 14 treatments in six weeks.

#### 2.2.3. Outcome Assessments

Trained investigators blinded to group allocation used an internationally standard assessment instrument, the Western Ontario and McMaster Universities osteoarthritis index (WOMAC) to determine treatment effectiveness [32]. Assessments were performed at baseline, after the initial treatment and at weeks 2, 6, and 10 after the initiation of treatment. The Chinese version, WOMAC LK3.1, consists of 24 items divided into 3 subscales: pain (5 items), stiffness (2 items), and physical function (17 items). If both knees were affected, the most painful knee was assessed [31, 33–35]. Adverse events were observed by laser technicians and documented at each visit.

### 2.3. Statistical Analysis

Since this study was a first step to evaluate the usefulness of TCM pattern diagnosis in the effectiveness study of this new laser treatment, we designed a relatively small pilot study with more intensive repeated measures per patients (for improving the power). The sample size requirement for each TCM pattern group is

(1) $$\begin{array}{c} n=\frac{2{\left(z_{\alpha /2}+z_{\beta }\right)}^{2}\left[1+\left(m-1\right)\rho \right]}{m\Delta^{2}},\;\text{where}\;\;\Delta =\frac{d}{\sigma }, \end{array}$$

*m* is the number of repeated measures per subject, Δ is the effect size, and *ρ* is the intercorrelation among repeated measures. Some reference values were estimated based on our previously reported clinical trials with this laser device [22, 23]. Since our current study is a pilot study for detecting the relative big signals, the effective size (Δ) for our study is 0.7. The intercorrelation *ρ* is about 0.8, *α* = 0.05, with the power (1 − *β*) of 80%. So, the sample size required for each group is 26. More sample size requirements for rationales with different setting of effect sizes (Δ) and *ρ* are listed in the appendix. To compare WOMAC score changes pre- and post-treatment, pair *t*-test was used. To determine the differences between the two TCM pattern groups, change from baseline was compared at each treatment using the equation [(Baseline  −  Post-treatment)/Baseline × 100%]. Normal distribution test and Levene's test for equality of variances for WOMAC scores of different groups and at different treatment stages were conducted to check their normality and equal variance assumptions. ANOVA was conducted to test difference among groups for each time point of assessment. Statistical significance was set that *P* value is below 0.05.

Multivariate linear regression for each time point was conducted to determine whether TCM pattern could influence treatment effect after controlling the confounding effects from age, gender, duration of disease, and number of affected knees. The rates of pain, stiffness, and function impairment reduction after treatment were the dependent variable; the other factors were the independent variables. Regression coefficients for TCM patterns are reported for each time points. 

## 3. Results

**Figure 2:**
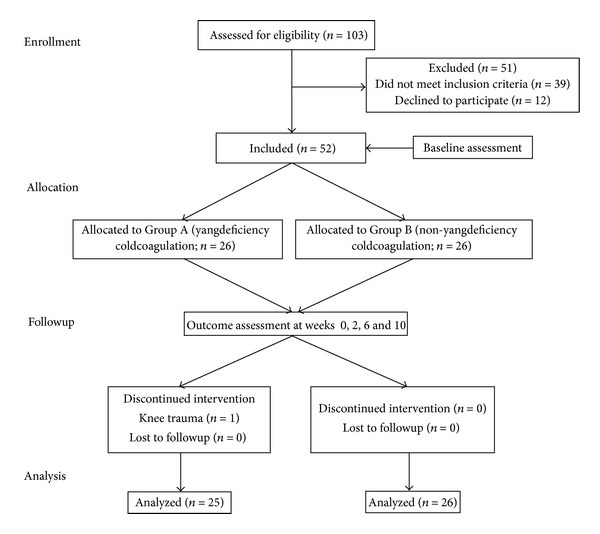
Flow chart of patient recruitment.

A total of 103 participants were assessed for eligibility. Fifty-two knee OA patients who met the inclusion/exclusion criteria were included in the study and allocated into the *yang* deficient (Group A) or the non-*yang* deficient group (Group B). One patient in Group A dropped out prior to the treatment due to a knee injury. None of the other participants was lost to followup (Figure 2). 

There was no statistically significant difference in the baseline characteristics of age, affected knees, and duration of disease between the two groups. Gender distribution was significantly different with more women in the *yang* deficient group than in the non-*yang* deficient group (Table 1).

Patients improved significantly in all three WOMAC score subcategories after week 2 of treatment in all patients combined (Table 2). There were no significant differences between the two groups in WOMAC baseline scores. At each time point of the outcome assessment, there was no statistically significant difference between the two groups in WOMAC pain or stiffness scores. However, WOMAC function scores of [Group A] (*yang* deficiency cold coagulation) significantly improved compared to those of [Group B] after initial treatment (*P* = 0.049), at week 2 (*P* = 0.046) and week 6 (*P* = 0.042; Table 3). No adverse events were observed in either group.

Regression analysis shows that at weeks 2, 6, and 10, WOMAC function score improvement is significantly associated with TCM pattern; pain and stiffness scores are not (Table 4).

Regression analysis also reveals positive association between symptom improvement and several other factors: the longer the duration of disease and the older the patient, the less improvement in pain and function; the more severe the pain, stiffness, and function impairment, the more effective the treatment (data not shown).

## 4. Discussion

In our previous two-arm randomized controlled trials, we found the combined 10.6 *μ*m–650 nm laser to be safe and significantly more effective on patients with knee OA than a sham control, as shown by WOMAC pain scores at week 2 [22, 23]. The results of the present trial are consistent with the data of those trials. The WOMAC pain score improvement rate at week 2 of the treatment reached 35%. A rate of 40% improvement was observed after 6 weeks of treatment, and improvement rose to 46.76% during the four-week followup after completion of the treatment (Table 2). 

Although randomized controlled trials (RCTs) are still the gold standard for evaluating the safety and efficacy of an intervention, their major limitation is that they might not reflect actual clinical practice [34]. In recent years, suggestions have been raised in the research community regarding the need for patient-centered, pragmatic research under real-life conditions in order to determine which intervention is optimal for which patient [36–38]. Traditional Chinese acupuncture practice often requires individualized treatment based on TCM pattern differentiation [39]. Consequently, treatment can vary from patient to patient and is often complex, involving several modalities such as acupuncture, moxibustion, cupping, or electrical stimulation. In the present study, we employed TCM pattern differentiation to determine whether thermal laser acupuncture is more effective on patients diagnosed with the TCM pattern yang deficiency and cold stagnation than on those with other TCM patterns.

Our results show that although patients in both groups markedly improved from baseline after the laser treatment, a clear trend indicates that more improvement in WOMAC subcategories of pain and stiffness among the *yang* deficient patients of Group A than among those in the non-*yang* deficient patients of Group B. In WOMAC function score, Group A significantly improved compared to Group B at almost all assessed time points (Table 3). Furthermore, our secondary regression analysis showed a significant positive correlation between *yang* deficiency and treatment effect in WOMAC function at all time points after week 2 (Table 4). These findings suggest that TCM pattern differentiation should guide the use of treatment techniques in acupuncture practice and might be important in designing future clinical trials with TCM interventions that might maximize treatment effect [40].

A major limitation of this study is that the patients in this trial were not randomized due to the nature of such study that may cause a selection bias. In order to minimize such bias, we separated TCM practitioner who made the TCM pattern diagnosis from those who performed laser treatments. To control this selection bias, multivariate linear regressions were used to control the potential confounding effects. In future study, we want to use some advanced longitudinal analysis approaches (e.g., random effect models) to evaluate the usefulness of TCM pattern diagnosis in the effectiveness assessment of this laser treatment, controlling not only the confounding effects from observed covariates but also the unobserved heterogeneity across patients. However, such mixed model approach was not carried on in current study due to technical limitation. It was our intention that this study was a first step to evaluate the usefulness of TCM pattern diagnosis in this effectiveness study which reflects daily TCM practice by individual practitioners. A future larger study is needed to confirm the findings of the present study. 

We are aware that the improvement rate in function is inconsistent with that of pain, a finding similar to that reported in a large clinical trial of acupuncture on knee OA, in which function but not pain improvement was observed after eight weeks of acupuncture treatment [31]. We speculate a possible reason for this inconsistency is that patients with improved function are likely to increase their physical activity, which might minimize their pain relief. Alternatively, the sample size of the present study might have been insufficient, particularly for a comparative outcome study in which data from two active treatment groups was compared [41]. 

It is interesting to note that more women are in yang deficient group than in the non-yang deficient group. While we cannot determine the reason for this difference, we speculate that this might be explained by TCM theory that women are overall a yin predominant pattern as compare to men who are generally more in yang predominant pattern. Alternative reason could be our small sample size so that this gender difference did happen by chance.

The mechanisms of laser irradiation have been widely studied in recent years [42, 43]. It has been reported that the 650 nm laser can penetrate the skin to between 10 and 25 mm [13, 17]. Research data also suggest that the significant pain relief in OA patients treated with low level lasers such as the 650 nm is the result of the laser's induction of neurotransmitters, which are important in endogenous pain modulation [44]. Because of its unique characteristics, which are similar to those of acupuncture needle treatment, 650 nm laser irradiation has been used as an alternative for acupuncture [22, 23]. In contrast to the low level laser, the CO_2_ laser radiation at 10.6 *μ*m wavelength, a far-infrared light, has a fairly persistent thermal effect [45] that can penetrate approximately 50 *μ*m into the body [46]. The radiant energy is absorbed by the superficial layers of the skin, resulting in a rise in skin temperature [47]. The thermal nature of this 10.6 *μ*m laser therefore is similar to the thermal effect of traditional moxibustion.

In conclusion, our study suggests that (1) TCM pattern differentiation, which has been widely practiced for centuries, should play an important role in TCM practice, and (2) thermal laser acupuncture at combined 10.6 *μ*m–650 nm wavelengths is safe and useful in treating *yang* deficient knee OA patients. Due to the small sample size and possible patient selection bias of this trial, a larger, randomized pragmatic clinical trial is warranted.

## Figures and Tables

**Table 1:** Baseline patient characteristics.

	Group A.* Yang *deficiency	Group B. No*n-yang *deficiency
	(*n* = 25)	(*n* = 26)
Age (years)	62.36	63.04
Gender (F/M)	20/5	14/12*
Affected knees (bilateral/single)	16/9	18/8
Durations (year)	8.08	7.46

*P < 0.05. There was no difference at baseline in group characteristics except for gender. There were more female patients in Group A.

**Table 2:** WOMAC score changes from baseline (Group A and Group B combined, *n* = 51).

	Pain	Stiffness	Function
	95% CI	95% CI	95% CI
First treatment	−4.98 [−9.38, −0.58]*	−4.27 [−9.34,0.79]	−2.12 [−5.63,1.39]
wk 2	−14.57 [−19.45, −9.70]**	−15.79 [−22.77, −8.82]**	−9.65 [−14.17, −5.12]**
wk 6	−15.29 [−20.63, −9.94]**	−17.76 [−24.04, −11.48]**	−11.75 [−16.61, −6.89]**
wk 10	−17.63 [−23.01, −12.25]**	−19.48 [−25.88, −13.08]**	−13.60 [−18.38, −8.81]**

*P value < 0.05 compared to baseline.

**P value < 0.001 compared to baseline.

**Table 3:** Percentage changes in WOMAC score values from baseline (mean ± SD) [(baseline − posttreatment)/baseline × 100%].

		Group A (diff %) (*n* = 25)	Group B (diff %) (*n* = 26)	*P* value	Total (diff %)
Pain	Initial treatment	8.98 ± 42.46	−3.07 ± 70.3	0.443	2.6 ± 58.1
	Week 2	37.72 ± 36.32	32.57 ± 40.63	0.634	35.09 ± 38.28
	Week 6	50.28 ± 35.55	30.3 ± 55.59	0.132	40.10 ± 47.47
	Week 10	51.25 ± 40.24	42.45 ± 54.95	0.516	46.76 ± 48.03

Stiffness	Initial treatment	−75.7 ± 358.31	−30.67 ± 110.22	0.552	−54.71 ± 260.86
	Week 2	−2.44 ± 125.39	−9.13 ± 136.97	0.856	−6.01 ± 130.02
	Week 6	30.01 ± 74.74	22 ± 67.94	0.691	25.11 ± 70.42
	Week 10	40.29 ± 55.50	18.59 ± 111.20	0.381	27.13 ± 89.36

Function	Initial treatment	10.93 ± 33.68*	−21.5 ± 73.00	**0.049**	−5.60 ± 58.96
	Week 2	36.18 ± 29.74*	12.25 ± 50.85	**0.046**	23.98 ± 43.16
	Week 6	47.67 ± 29.67*	24.15 ± 48.32	**0.042**	35.68 ± 41.61
	Week 10	51.52 ± 34.66	33.15 ± 42.71	0.098	42.15 ± 39.68

*P value < 0.05 compared to Group B.

**Table 4:** Influence of TCM pattern on WOMAC score improvement rate from baseline using multivariate regression analysis controlling confounding.

	Pain/TCM			Stiffness/TCM			Function/TCM		
	Coefficient	SE	*P*	Coefficient	SE	*P*	Coefficient	SE	*P*
1st treatment	−0.134	0.169	0.432	0.115	0.791	0.885	−0.296	0.17	0.089
wk 2	−0.131	0.102	0.205	−0.28	0.401	0.488	−0.27	0.118	0.027*
wk 6	−0.257	0.132	0.057	−0.171	0.216	0.432	−0.293	0.111	0.012*
wk 10	−0.15	0.139	0.288	−0.408	0.260	0.123	−0.265	0.108	0.019*

*P < 0.05 indicates a positive correlation between the *yang* deficiency cold coagulation pattern and treatment effect.

**Table 5:** Sample sizes for rationales of different effect sizes (Δ) and *ρ*.

*ρ*	Δ (%)			
	50%	60%	70%	80%
0.5	34	24	18	14
0.6	40	28	21	16
0.7	46	32	23	18
0.8	51	36	**26**	20
0.9	57	40	29	23

## Appendix

See Table 5.
